# Spinal Cord Ischemia Following Thoracoabdominal Aortic Aneurysm Repair: Translational Insights from Stroke and Traumatic Injury for Biomarker Development

**DOI:** 10.3390/biomedicines14051144

**Published:** 2026-05-18

**Authors:** James A. Kelly, Miranda Witheford, Kong Teng Tan, Tiam Feridooni, Daniyal Mahmood, Carmen Garcia-Mere, Thomas F. Lindsay

**Affiliations:** 1 Department of Vascular Surgery, University Health Network, University of Toronto, Toronto, ON M5G 2C4, Canada; miranda.witheford@uhn.ca (M.W.); dan.mahmood@mail.utoronto.ca (D.M.); carmen.garciamere@uhn.ca (C.G.-M.); thomas.lindsay@uhn.ca (T.F.L.); 2 Department of Radiology, University Health Network, University of Toronto, Toronto, ON M5G 2C4, Canada; kongteng.tan@uhn.ca; 3 Division of Vascular Surgery, Schulich Heart Centre, Sunnybrook Health Sciences Centre, Toronto, ON M4N 3M5, Canada; tiam.feridooni@sunnybrook.ca

**Keywords:** spinal cord ischemia, thoracoabdominal aortic aneurysm, spinal cord perfusion pressure, biomarkers, neuroinflammation, ischemia–reperfusion injury, AQP4

## Abstract

**Background:** Spinal cord ischemia (SCI) is a severe complication of thoracoabdominal aortic aneurysm (TAAA) repair, associated with substantial morbidity and mortality. Despite advances in operative techniques, its pathophysiology remains incompletely understood, with no reliable biomarkers available for early detection or risk stratification. **Methods:** This narrative review synthesizes current evidence on the pathophysiology of SCI following aortic intervention, integrating insights from ischemic stroke and traumatic spinal cord injury to identify key mechanistic pathways and potential biomarker targets. **Results**: SCI results from multifactorial impairment of spinal cord perfusion pressure (SCPP) driven by extensive aortic coverage, disruption of segmental arterial inflow, hypotension, and impaired collateral circulation. While acute hypoperfusion initiates injury, secondary processes—including excitotoxicity, oxidative stress, and neuroinflammation—drive progression. Cytokine signaling and immune activation contribute to blood–spinal cord barrier disruption and vasogenic edema, with Aquaporin-4 playing a central role in delayed injury. Candidate biomarkers, including neuron-specific enolase, S100β, and glial fibrillary acidic protein, reflect neuronal damage but lack sufficient sensitivity and temporal resolution for clinical use. Emerging evidence supports a multimodal biomarker approach incorporating inflammatory, structural, and Aquaporin-4-dependent edema-related pathways. **Conclusions:** Spinal cord ischemia following thoracoabdominal aortic aneurysm repair is a dynamic and multifactorial process in which reduced spinal cord perfusion pressure represents a final common pathway linking diverse perioperative factors to ischemic injury. Secondary mechanisms, particularly neuroinflammation and Aquaporin-4-driven vasogenic edema, play a central role in injury propagation and represent promising targets for biomarker development. Future strategies should focus on longitudinal, multimodal biomarker approaches to improve early detection, risk stratification, and therapeutic intervention.

## 1. Introduction

Postoperative spinal cord ischemia (SCI) is a devastating complication of thoracoabdominal aortic aneurysm (TAAA) repair, manifesting as paraplegia, paraparesis, or incontinence [1,2,3], and associated with increased 30-day mortality and reduced long-term survival [4,5,6,7]. SCI arises from interruption of segmental arterial inflow, as TAAA repair necessitates coverage of intercostal and/or lumbar vessels supplying the spinal cord. This risk is present across all operative strategies, including open, endovascular, and hybrid approaches [1].

The societal and economic burden is substantial, with the costs estimated at $518,904 in the first year and $68,739 annually thereafter in the United States [8,9]. The expansion of complex fenestrated and branched endovascular repair (F/BEVAR) has broadened treatment eligibility to higher-risk patients, thereby increasing the population at risk for SCI. Reported incidence varies due to heterogeneity in definitions, but a systematic review estimates rates of approximately 13.5% following endovascular repair and 7.4% after open surgery [10].

**Figure 1:**
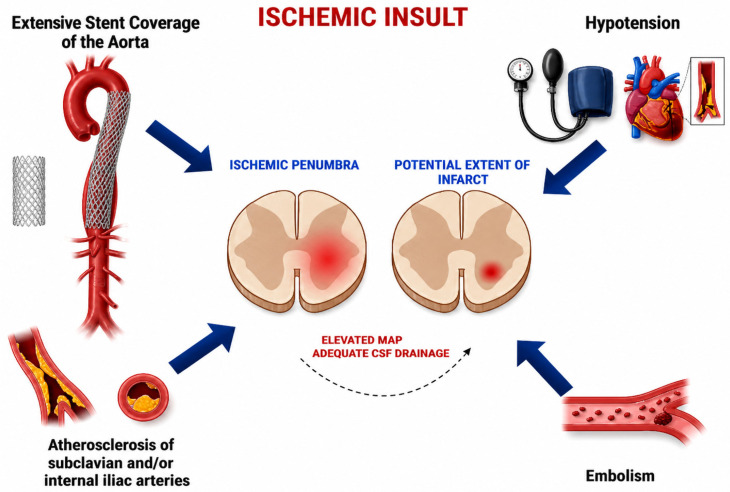
Multifactorial pathogenesis of spinal cord ischemia following thoracoabdominal aortic aneurysm repair, illustrating the central role of reduced spinal cord perfusion pressure (SCPP) as the final common pathway linking perioperative risk factors to ischemic injury.

The pathogenesis is multifactorial (Figure 1). The spinal cord is supplied by the anterior and posterior spinal arteries, interconnected via the vasocorona and supported by segmental inflow from vertebral, intercostal, lumbar, and internal iliac arteries [11,12]. Greater aortic coverage disrupts more segmental vessels and is associated with increased risk of ischemic injury [13,14]. A retrospective series of 142 non-ruptured TAAA repairs identified extent of aortic coverage—particularly of the thoracic segment—as the strongest predictor of SCI, demonstrating an exponential relationship with risk [15]. However, anatomic interruption alone does not fully explain SCI, as cases have been reported even with limited aortic coverage [16,17]. Additional risk factors include impaired baseline renal function (eGFR < 30) and intraoperative hypotension, particularly sustained systolic blood pressure < 90 mmHg for more than 15 min [18,19].

To date, biomarkers for pre-operative risk stratification of paraplegia have been explored in both serum and cerebrospinal fluid, but with limited success [20,21,22,23]. Currently, intraoperative neurological monitoring relies primarily on motor evoked potentials (MEPs); however, these are invasive, require specialist expertise, and lack consensus for routine use in open or endovascular thoracoabdominal aortic aneurysm (TAAA) repair [24]. As such, there remains no reliable pre- or intra-operative method to predict an individual patient’s risk of postoperative motor impairment [24].

Early identification of high-risk patients would enable improved risk stratification and more informed surgical decision-making. However, the absence of predictive biomarkers reflects an incomplete understanding of the pathogenesis of spinal cord ischemia (SCI) following TAAA repair. Insights from other central nervous system injuries, including stroke and traumatic spinal cord injury, may offer translational opportunities in relation to ischemia–reperfusion injury. Immune responses of T cells and microglia/macrophages demonstrate a dynamic balance of pro- and anti-inflammatory signaling, which may be either protective or deleterious depending on timing and tissue context [25].

Mechanistically, SCI following aortic repair arises from disruption of segmental arterial inflow and spinal collateral networks [14]. This results in reduced spinal cord perfusion pressure (SCPP), defined as the difference between mean arterial pressure and cerebrospinal fluid (CSF) pressure, predisposing to ischemia. Immediate SCI is typically driven by acute hypoperfusion and failure of collateral circulation, whereas delayed SCI reflects a more complex interplay of reperfusion injury, edema, and evolving microvascular dysfunction.

Similar to that of stroke and traumatic spinal cord injury, current management of SCI focuses on preserving the ischemic penumbra through optimization of perfusion and oxygen delivery [26]. Biomarker interpretation must account for the distinct pathophysiologic context of aortic intervention, where both immediate and delayed injury patterns are observed. A deeper understanding of these mechanisms may help identify novel biomarkers and therapeutic targets. Accordingly, the aim of this paper is to review the pathophysiologic mechanisms and inflammatory pathways underlying SCI.

## 2. Literature Search Approach

A narrative literature review was conducted using PubMed/MEDLINE and Embase databases to identify relevant experimental, translational, and clinical studies relating to spinal cord ischemia following thoracoabdominal aortic aneurysm repair. Additional research relating to ischemic stroke, traumatic spinal cord injury, neuroinflammation, excitotoxicity, edema biology, and biomarker development was also reviewed to provide translational context. Search terms included combinations of “spinal cord ischemia,” “thoracoabdominal aortic aneurysm,” “aortic repair,” “biomarkers,” “neuroinflammation,” “stroke,” “traumatic spinal cord injury,” “microglia,” “macrophages,” “T-cells,” and “aquaporin-4.”

Reference lists of selected articles were additionally screened to identify further relevant studies. Given the heterogeneity of the available literature and the translational focus of the review, a narrative synthesis approach was employed rather than a formal systematic review methodology.

## 3. Astroglial Injury Markers

Neuron-specific enolase (NSE), glial fibrillary acidic protein (GFAP), and S100β are among the earliest and most extensively studied biomarkers in the context of spinal cord ischemia. NSE, a glycolytic enzyme localized to neurons, is generally considered a marker of neuronal injury, whereas GFAP and S100β predominantly reflect astroglial injury and disruption of the blood-spinal cord barrier. Their biological relevance is supported by the pathophysiology of spinal cord ischemia, which involves neuronal loss, astrocytic activation, and barrier dysfunction; however, their clinical utility remains limited.

Early clinical studies in TAAA surgery demonstrated that cerebrospinal fluid may provide a more sensitive environment to detect spinal cord injury than serum markers [23]. CSF S100β and lactate levels demonstrated stronger correlation with clinically evident spinal cord ischemia than those in serum [22]. These findings supported the concept that intrathecal biomarkers may better reflect localized spinal cord injury although their application is constrained by the invasiveness of CSF sampling and the relatively low incidence of clinical events.

More recent studies have evaluated NSE, GFAP, and S100β in combination [27] in patients undergoing open and endovascular TAAA repair. This demonstrated associations between biomarker elevation and neurophysiologic changes suggestive of cord ischemia, although actionable thresholds could not be established. Similarly, pilot data from complex endovascular aortic repair suggest that multiple CSF biomarkers, including GFAP, neurofilament light, and tau, may show marked elevation in patients who develop spinal cord ischemia compared with those who do not. This supports the concept that multi-marker panels may provide greater discriminatory value than individual analytes [27].

Two key limitations arise from the existing literature. First, many studies are limited by sample sizes and a low incidence of clinically overt spinal cord ischemia, reducing statistical power and limiting the ability to define reliable diagnostic thresholds. Secondly, the specificity of these biomarkers—particularly GFAP and S100β—is limited. While they reflect astroglial injury, they are not specific to spinal cord ischemia and may be elevated in a range of other conditions, including traumatic brain injury, ischemic stroke, subarachnoid hemorrhage, and systemic inflammatory states [28]. NSE is similarly affected by extracranial sources and hemolysis, further complicating interpretation. Collectively, these limitations highlight that NSE, GFAP, and S100β are unlikely to function as standalone diagnostic biomarkers.

## 4. The Inflammatory Cascade and Excitotoxicity: Biomarker Opportunities in SCI

Neurologic injury in spinal cord ischemia (SCI) occurs both at the time of ischemic insult and during reperfusion, with inflammation propagating injury rostrally and caudally from the initial site [29,30,31]. Ischemia triggers rapid upregulation of pro-inflammatory cytokines and chemokines, including IL-1α, IL-6, IL-8, and TNF-α [32], as well as arachidonic acid metabolites [33], promoting expression of endothelial adhesion molecules [34]. These facilitate leukocyte recruitment via receptors such as L-selectin, lymphocyte function-associated antigen-1, and VLA-4 [35]. Subsequent neutrophil infiltration and phagocytosis generate reactive oxygen species (ROS), amplifying oxidative injury [36].

A central mechanism linking ischemia to neuronal death is excitotoxicity. Cellular ATP depletion impairs Na^+^/K^+^-ATPase function, disrupting ionic homeostasis and causing membrane depolarization, intracellular sodium accumulation, and cellular swelling [37]. This is followed by activation of voltage-gated calcium channels and calcium influx. Concurrently, glutamate—the primary excitatory neurotransmitter—activates NMDA and AMPA receptors, further increasing intracellular calcium and promoting neuronal injury [38].

**Figure 2:**
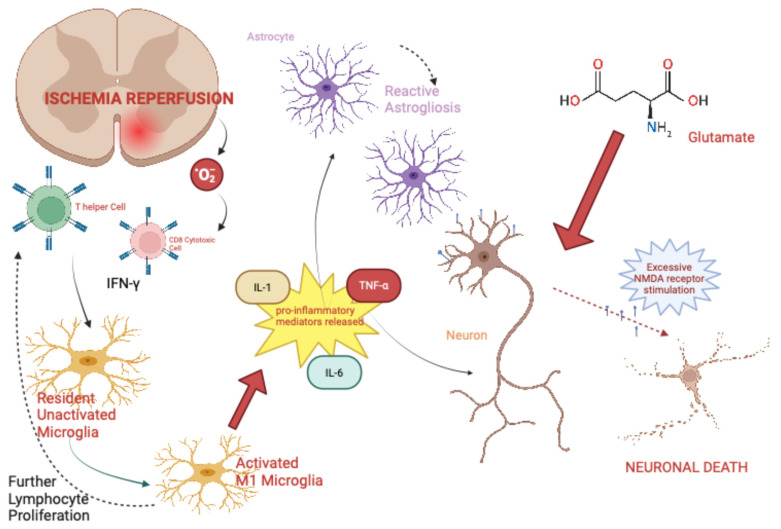
Mechanistic model of excitotoxic injury in spinal cord ischemia, illustrating the interaction between neuroinflammation, microglial/macrophage activation, and reactive astrogliosis in promoting glutamate release and impaired uptake. Excess extracellular glutamate activates AMPA and NMDA receptors, leading to calcium influx, neuronal injury, and propagation of secondary damage.

Neuroinflammation then amplifies this process. Infiltrating immune cells, including neutrophils, macrophages, and microglia [39], alongside Th1 lymphocyte-derived interferon-γ, stimulate TNF-α release via MEK/ERK signaling pathways [40]. TNF-α activation of TNF-R1 receptors on microglia and astrocytes promotes additional glutamate release [41,42], perpetuating a cycle of excitotoxicity and neuronal death (see Figure 2).

Given this mechanistic framework, excitotoxicity has been a major therapeutic target, particularly in stroke, and could be of relevance in spinal cord ischemia. NMDA receptor antagonism has demonstrated neuroprotection in pre-clinical stroke models but has failed to translate into consistent clinical benefit [42,43]. Several factors likely underlie this discrepancy. Experimental models typically involve controlled, homogeneous ischemic insults, whereas human disease is heterogeneous, with variable timing, collateral circulation, and comorbidities influencing injury evolution. In addition, NMDA receptor signaling is not solely deleterious; it also plays a role in normal synaptic function and neuronal survival, and broad antagonism may disrupt physiological processes [42]. Critically, the therapeutic window for effective NMDA inhibition appears narrow, and delayed administration—common in clinical settings—may occur after irreversible injury has developed. For example, the ENACT trial evaluating NA-1 in patients undergoing endovascular cerebral aneurysm repair demonstrated an acceptable safety profile but failed to show clinical efficacy despite promising preclinical results [44].

Despite these translational disappointments within human cerebral models, these mechanistic pathways still highlight potential biomarker targets within the remit of cord ischemia. Cytokines such as IL-6 and TNF-α may reflect early inflammatory activation, while markers of neuronal injury (e.g., NSE, GFAP) and excitotoxic signaling may indicate downstream cellular damage.

## 5. Immune Cell Phenotypes in Spinal Cord Injury

Immune cells are central mediators of secondary injury and repair following central nervous system (CNS) insults [43]. Macrophage and microglial responses have traditionally been described using the simplified M1/M2 polarization framework, in which M1 phenotypes are considered pro-inflammatory and M2 phenotypes reparative. However, this binary classification is now recognized as an oversimplification. Contemporary transcriptomic and single-cell analyses demonstrate that macrophages and microglia exist along a dynamic continuum of activation states, shaped by temporal factors, the local tissue environment, metabolic signaling, and interactions with other immune and neural cells. Rather than discrete phenotypes, these cells adopt overlapping programs encompassing inflammatory, phagocytic, reparative, and remodeling functions that evolve throughout the course of injury [45].

In spinal cord ischemia this temporal plasticity is relevant. Early immune activation contributes to secondary injury through the release of pro-inflammatory cytokines, oxidative stress, and disruption of the blood spinal cord barrier. However, these same responses are essential for clearance of necrotic debris and initiation of tissue repair. As the injury evolves, immune cells may adopt more reparative functions, akin to the so-called M2 phenotype, supporting angiogenesis, extracellular matrix remodeling, and resolution of inflammation [46]. Dysregulation of these processes, however, can perpetuate inflammation, promote gliosis, and inhibit functional recovery. Consequently, therapeutic strategies aimed at broadly suppressing inflammation or promoting a single macrophage phenotype may be overly reductive; instead, there is a need to understand and modulate context-specific immune responses across different phases of injury [47].

Translation of these findings into ischemic spinal cord injury remains limited, with much evidence derived from traumatic models. Pre-clinical studies have demonstrated that modulation of immune responses—through approaches such as mesenchymal stem cell therapy or alteration of extracellular matrix signaling—can shift macrophage activity toward more reparative states and improve functional outcomes [48,49,50,51,52,53,54]. However, the extent to which these findings apply to ischemic injury, particularly in the context of thoracoabdominal aortic aneurysm repair, is not yet well defined.

Human data remain sparse but suggest that inflammatory signaling is closely linked to injury severity and recovery; again, limited by a lack of studies utilizing relevant biological samples. In patients with traumatic spinal cord injury, cerebrospinal fluid (CSF) levels of cytokines such as IL-1, IL-8, and monocyte chemoattractant protein-1 (MCP-1), as well as structural proteins including glial fibrillary acidic protein (GFAP), S100β, and Tau, correlate with injury severity and may predict neurological recovery [55]. Similarly, in patients undergoing open thoracic or thoracoabdominal aortic repair, elevated CSF IL-8 has been associated with the development of paraplegia [56]. These findings suggest that CSF reflects the local neuroinflammatory environment and represents a promising source for biomarker discovery.

Importantly, inflammatory activation is closely coupled to disruption of the blood–spinal cord barrier and the development of spinal cord edema. In this context, Aquaporin-4 (AQP4), a key regulator of water transport in astrocytic end-feet, plays a central role in mediating edema formation. Upregulation of AQP4 following ischemia may exacerbate cord swelling, increase intramedullary pressure, and further compromise microvascular perfusion, thereby amplifying secondary injury. This mechanistic link between immune activation, barrier dysfunction, and edema formation highlights the need for integrated biomarker strategies that capture multiple aspects of injury biology. The role of AQP4 as a potential biomarker of cord ischemia is discussed further below. Taken together, these data support a shift away from static models of immune activation toward a dynamic, systems-level understanding of neuroinflammation in spinal cord ischemia. Biomarker approaches that incorporate inflammatory mediators, structural injury markers, and edema-related pathways may provide greater insight into injury progression and offer opportunities for improved risk stratification and therapeutic targeting.

## 6. Role of T Cells in Stroke and Application to Spinal Cord Ischemia

Adaptive immune responses, particularly those mediated by T lymphocytes, have emerged as important contributors to secondary injury following central nervous system ischemia. Experimental stroke models demonstrate that T cells exert both deleterious and protective effects depending on subtype, timing, and local inflammatory context. In murine models of middle cerebral artery occlusion, T cell deficiency in SCID and Rag1^−^/^−^ mice is associated with reduced infarct volume and attenuated inflammatory injury, implicating T cells as key mediators of ischemic damage [57]. This effect appears largely independent of B cells, as reconstitution of B cells alone does not reverse neuroprotection. More targeted studies have further demonstrated that depletion of CD8^+^ T cells reduces infarct size, while deficiency of chemokines such as RANTES (CCL5), which facilitate T cell recruitment to sites of injury, similarly confers protection [58,59].

In contrast, regulatory and anti-inflammatory T cell subsets may exert neuroprotective effects. Regulatory T cells and Th2-associated cytokine signaling appear to modulate excessive inflammation and limit secondary tissue injury, with depletion of regulatory T cells or deficiency of IL-4 resulting in larger infarct volumes in experimental stroke [60]. These findings highlight the dualistic nature of T cell responses in ischemic injury, in which distinct lymphocyte populations may either exacerbate or attenuate tissue damage.

Spatial and temporal dynamics further refine this paradigm. T cells preferentially accumulate within peri-infarct regions containing viable but vulnerable neurons rather than the irreversibly damaged ischemic core [61,62]. This region, often referred to as the “inflammatory penumbra,” represents an area in which secondary immune-mediated injury may remain potentially reversible. Although this concept has not been directly established in spinal cord ischemia following thoracoabdominal aortic aneurysm repair, it provides a compelling mechanistic framework for understanding delayed neurologic deterioration after aortic intervention.

Mechanistically, T cells contribute to secondary injury through cytokine release, amplification of innate immune signaling, and disruption of blood–central nervous system barrier integrity. Endothelial activation following ischemia promotes expression of adhesion molecules such as ICAM-1 and VCAM-1, facilitating T cell adhesion and transmigration into injured tissue [63]. Once within the parenchyma, activated T cells release pro-inflammatory cytokines, including interferon-γ (IFN-γ), which stimulate microglial and macrophage activation and propagate inflammatory signaling cascades. This process contributes to blood–spinal cord barrier dysfunction, increased vascular permeability, and vasogenic edema.

Importantly, extrapolation of these findings from cerebral ischemia to spinal cord ischemia should be approached cautiously. The spinal cord possesses distinct vascular and collateral anatomy, with greater dependence on segmental arterial inflow and increased susceptibility to hypoperfusion. Differences in blood–spinal cord barrier characteristics, cytokine expression profiles, and immune cell trafficking may further influence inflammatory responses within spinal cord tissue. Limited experimental data suggest that ischemic spinal cord injury may exhibit a heightened pro-inflammatory milieu characterized by increased expression of Th1-associated cytokines such as IFN-γ, although direct human data remain limited. Collectively, these findings support a more nuanced understanding of T cell biology in spinal cord ischemia, in which lymphocyte responses exist along a functional spectrum rather than discrete phenotypic states. While cytotoxic and Th1-associated responses may amplify secondary injury, regulatory and anti-inflammatory T cell populations may contribute to resolution of inflammation and tissue repair. From a translational perspective, T cell associated cytokines and chemokines, may represent promising candidates for multimodal biomarker development in spinal cord ischemia following thoracoabdominal aortic aneurysm repair.

## 7. Aquaporin-4 (AQP4) and Edema as Biomarker Pathways in SCI

Vasogenic edema represents a key mechanism of secondary injury in spinal cord ischemia, contributing to increased intramedullary pressure, and further impairment of microvascular perfusion. AQP4, a water channel highly expressed in astrocytic end-feet at the blood–brain and blood–spinal cord barriers, plays a central role in regulating water flux in response to ischemic and inflammatory stimuli [64].

Emerging experimental evidence supports a critical role for AQP4 in edema formation following ischemic injury. Hypoxia has been shown to upregulate AQP4 expression [65], promoting water influx into astrocytes and contributing to cytotoxic edema in animal models of cerebral ischemia. In AQP4-null mice, reduced edema formation and improved survival have been demonstrated following ischemic insult [66], while pharmacologic inhibition of AQP4 has been associated with attenuation of cerebral edema [67]. Perivascular localization of AQP4 has also been proposed as a key regulator of water flux during reperfusion injury [68]. Similar findings have been observed in models of spinal cord trauma, where AQP4-mediated water influx contributes to cord swelling and secondary injury, and its inhibition mitigates edema formation. Together, these data highlight a conserved role for AQP4 in mediating water homeostasis across central nervous system injury states.

In the context of spinal cord ischemia, these mechanisms converge on disruption of the blood–spinal cord barrier and progressive edema formation. Neuroinflammatory signaling, including cytokines such as IL-6 and TNF-α, promotes endothelial dysfunction and increased vascular permeability, facilitating fluid extravasation into the spinal cord parenchyma. Upregulation of AQP4 in response to these signals may exacerbate water accumulation, amplifying cord swelling and increasing intramedullary pressure, thereby further compromising spinal cord perfusion. This interaction between inflammation, barrier dysfunction, and AQP4-mediated edema is particularly relevant in delayed SCI, where progressive swelling and impaired venous drainage contribute to neurological deterioration hours to days after the index procedure [25,66].

Importantly, the role of AQP4 may be temporally dynamic. While early upregulation appears to facilitate edema formation, AQP4 may also contribute to later phases of edema resolution through clearance of interstitial fluid. This dual role underscores the complexity of targeting AQP4 therapeutically and highlights the importance of temporal context when interpreting its function in SCI.

From a translational perspective, AQP4-associated pathways represent both a potential therapeutic target and a candidate biomarker of secondary injury [69]. In a recent exploratory study, CSF AQP4 concentrations > 15 ng/mL demonstrated high specificity for irreversible paraplegia following endovascular TAAA repair [69]. Given its central role in edema formation and its integration with inflammatory and vascular processes, AQP4 may serve as an indirect indicator of evolving spinal cord injury, particularly in delayed presentations. Integration of AQP4-related signaling with markers of neuronal injury and inflammation may enhance the sensitivity of multimodal biomarker strategies.

Collectively, AQP4 can be conceptualized as a key mechanistic node linking neuroinflammation, blood–spinal cord barrier dysfunction, and edema in spinal cord ischemia [70]. Targeting this pathway—and capturing its activity through biomarker approaches—may provide a promising avenue for improving both the detection and management of SCI following thoracoabdominal aortic aneurysm repair.

## 8. The Future for Biomarkers in Spinal Cord Ischemia

Future biomarker strategies in spinal cord ischemia will likely require a shift away from isolated static analytes toward longitudinal, multimodal approaches capable of capturing the evolving biology of injury. Although many mechanistic insights derive from experimental stroke and traumatic spinal cord injury models, direct human evidence in ischemic spinal cord injury following aortic intervention remains limited. Consequently, several pathways discussed should be interpreted as biologically plausible translational frameworks rather than established mechanisms in clinical SCI after TAAA repair. Moreover, although many mechanistic insights are derived from the stroke and traumatic spinal cord injury literature, important differences in vascular anatomy and injury kinetics may influence their applicability to ischemic spinal cord injury following aortic intervention.

Previous biomarker studies have been limited by small sample sizes, low event rates, inconsistent access to biologically relevant samples such as CSF, and reliance on isolated analytes that inadequately reflect the temporal complexity of ischemic and inflammatory injury (Table 1). Dynamic changes in biomarker trajectories may ultimately prove more informative than isolated measurements at single time points. Table 1 below is a summary of the most investigated biomarkers and their respective limitations. An ideal biomarker platform in this setting would integrate markers of neuronal injury, neuroinflammation, and edema-related pathways with physiologic and neurophysiologic monitoring to distinguish early hypoperfusion from delayed secondary injury. Such approaches, which have been adopted more recently with the description of edema-based insults, may ultimately provide greater sensitivity for early detection, improved risk stratification, and more precise therapeutic targeting following thoracoabdominal aortic aneurysm repair.

## 9. Conclusions

To date, no cerebrospinal fluid or serum biomarker has demonstrated sufficient sensitivity or temporal resolution for the early detection of spinal cord ischemia following thoracoabdominal aortic aneurysm repair. Progress in this field has been limited by small sample sizes, restricted access to biological specimens, and the relatively low incidence of clinically overt neurologic injury. As the use of endovascular and hybrid techniques expands, the population at risk for SCI will increase, yet traditional randomized trial designs may remain impractical.

Advancing this field will require a translational approach grounded in mechanistic understanding. Insights from ischemic stroke and traumatic spinal cord injury highlight the importance of secondary injury pathways, including neuroinflammation, excitotoxicity, and edema. Emerging evidence suggests that adaptive immune responses, particularly T cell-mediated signaling, alongside the dynamic continuum of monocyte/macrophage activation, contribute to the evolving inflammatory milieu observed after aortic intervention. These processes are closely linked to disruption of the blood–spinal cord barrier and the development of spinal cord edema, with mediators such as AQP4 representing a key interface between inflammation and secondary injury.

A deeper understanding of these interconnected pathways is essential for identifying clinically meaningful biomarkers. Future strategies should focus on multimodal biomarker approaches that integrate inflammatory mediators, structural injury markers, and edema-related pathways, combined with longitudinal sampling and correlation with clinical and neurophysiologic data. Such approaches may enable earlier detection of injury, improved risk stratification, and the development of targeted therapies. Ultimately, bridging the gap between mechanistic insight and clinical application represents a critical step toward reducing the burden of paraplegia following TAAA repair.

## Figures and Tables

**Table 1:** Summary of the most investigated biomarkers of spinal cord ischemia post thoracoabdominal aortic aneurysm repair, and their respective limitations.

Biomarker	Pathway	Source	Potential Role	Key Limitation
NSE	Neuronal injury	CSF/serum	Early injury	Poor specificity
GFAP	Astroglial injury	CSF	Structural injury	CNS non-specific
IL-6	Inflammation	CSF	Early inflammatory activation	Temporal variability
AQP4	Edema	CSF	Delayed injury	Preliminary data
Glutamate	Excitotoxicity	CSF	AMPA/NMDA stimulation	Preliminary data

## Data Availability

No new data were created or analyzed in this study.
